# A multi-criteria decision analysis (MCDA) tool for purchasing off-patent oncology medicines in Egypt

**DOI:** 10.1186/s40545-022-00414-2

**Published:** 2022-03-01

**Authors:** Baher Elezbawy, Ahmad Nader Fasseeh, Amal Sedrak, Randa Eldessouki, Mary Gamal, Mariam Eldebeiky, Hanaa Amer, Shimaa Akeel, Ahmad Morsy, Amira Amin, Amr Shafik, Sherif Abaza, Zoltán Kaló

**Affiliations:** 1 Syreon Middle East, Alexandria, Egypt; 2 Eötvös Loránd University, Budapest, Hungary; 3 The Egyptian Authority for Unified Procurement, Medical Supply, and The Management of Medical Technology (UPA), Cairo, Egypt; 4 Fayoum University, Fayoum, Egypt; 5 Ain Shams University, Cairo, Egypt; 6 Presidential Program for Women’s Health, Ministry of Health and Population (MoHP, Cairo, Egypt; 7 Egyptian Pharmaceutical Vigilance Center, Egyptian Drug Authority (EDA), Cairo, Egypt; 8 Syreon Middle East, Cairo, Egypt; 9 Semmelweis University, Budapest, Hungary; 10 Syreon Research Institute, Budapest, Hungary

**Keywords:** Off-patent pharmaceuticals, Multi- criteria decision analysis, Purchasing, Oncology medicines, Tendering process, MCDA

## Abstract

**Background:**

Multi- criteria decision analysis (MCDA) can assist policymakers in objectively choosing between alternative therapeutic options based on multiple value attributes. Our aim was to create an MCDA tool for the national tenders of off-patent oncology medicines in Egypt.

**Methods:**

An initial list of criteria was developed through a literature review complemented by local expert interviews. Price or cost-related criteria were excluded to abide by the national regulations of the tender process. Next, a workshop hosting diversified stakeholders representing different governmental bodies was held. Anonymous voting was used to rank and weigh the criteria as well as assigning scores. Price was added as a separate step to identify best option based on price per point. The tool was then tested on a national tender sample of off-patent oncology medicines to assess its performance, and it was readjusted accordingly in a second workshop.

**Results:**

Seven non-price criteria were selected, including use in reference countries (23.49% weight), equivalence with the reference product (18.79%), manufacturing quality (15.53%), provision of pharmacovigilance services (12.94%), supply reliability (10.78%), previous use in local settings (9.8%) and macroeconomic benefit (8.67%).

A medicine receives a score ranging from 0 to 100% of each criterion’s weight. The aggregated score is calculated on a hundred-point scale. Based on participants’ consensus, an overall score of 65 was set as a cut-off for passing the technical eligibility phase of the tendering process. Any product receiving a lower score would be disqualified from the tender. For qualified products, the lower price per point represents preferential option for the national tender.

**Conclusions:**

The created MCDA tool is capable of objectively comparing similar off-patent oncology medicines by considering multiple value attributes and providing reliable scoring functions for each.

## Background

Multiple criteria decision analysis (MCDA) is increasingly used in healthcare to support evidence-based selection between alternative health technologies based on multiple mutually exclusive criteria [1, 2].

Generic pharmaceuticals are widely used whenever available. They are the first line of treatments for many chronic conditions to reduce healthcare costs [3, 4]. Pharmaceutical expenditure represents a significant proportion of the total health care expenditure in Egypt (34%) and is estimated as 67 billion EGP annually [5]. Therefore, better decisions related to selection between alternative pharmaceuticals could have a significant positive impact.

Efficient off-patent pharmaceutical policies are essential in maintaining the financial sustainability of the health care system, and enable patient access to medicines, especially in emerging economies with limited health care resources [6]. Price is an important component in purchasing off-patent medicines; however, making decisions solely based on one criterion may result in inefficient resource allocation. Disregarding other criteria (e.g., quality, supply reliability or pharmacovigilance) may lead to opposite results than those intended by the public pharmaceutical policy for off-patent pharmaceuticals. Decision-makers aim to either improve population health from the same budget or achieve cost containment without compromising health outcomes [7]. Either way, an evidence-based systematic approach for decision-making is required. Recently, MCDA is gaining momentum across several jurisdictions for assessing the value of off-patent drugs and to support public policy decisions [8–13].

Egypt is in the midst of a health system reform that aims to achieve universal health care coverage by 2030. Currently, the Egyptian healthcare system consists of a wide range of public healthcare providers, and financing agents. The procurement of drugs and medical devices used to be decentralized among the different health care providers, but recently, the Egyptian Authority for Unified Procurement, Medical Supply, and The Management of Medical Technology (UPA) was established to achieve bulk purchasing and ensure provision of high-quality health technologies at affordable prices to all the governmental entities, entitled by law 151/2019 [14]. Although the centralization of procurement provides major advantages due to the higher negotiation power and avoiding work duplication, it comes at a cost, where the impact of sub-optimal decisions affects all beneficiary entities. The UPA considers using MCDA as an evidence-based decision-making tool to evaluate and rank off-patent pharmaceuticals objectively, improve transparency, and increase consistency in decisions.^vi^.

The utilization of MCDA tools in Egypt for tendering purposes was lately enabled by the tender law 182 for the year 2018, and it was recently empowered by UPA establishment under the Act No. (151) of the year 2019, and its regulation No (777) [15, 16]. The new law allows the use of a scoring system in tenders,xvi which provides a legal framework for using the tool in tendering decisions.

Several versions of MCDA tools for off-patent medicines assessment exist [11] however, transferability of value assessment across jurisdictions is limited [17]. For instance, price (i.e., acquisition cost) is a common criterion in MCDA tools used for purchasing decisions [18], but it could not be included in the MCDA tool in Egypt, since the Egyptian tendering process has two separate phases. The first phase is the technical assessment of the medicine irrespective of the proposed price. Subsequently, the financial committee in the second phase reviews the report of the technical committee, and negotiates with the pharmaceutical companies accordingly, to select the best proposed price. As MCDA tools cannot be cloned from another country or setting, a tailored MCDA tool must be composed to support this specific selection process.

Developing countries with limited resources, like Egypt, need to develop their own MCDA tools for choosing between the available alternatives [19]. Our aim was to create an MCDA tool to be used by the UPA for purchasing off-patent oncology medicines.

## Methods

**Fig. 1:**
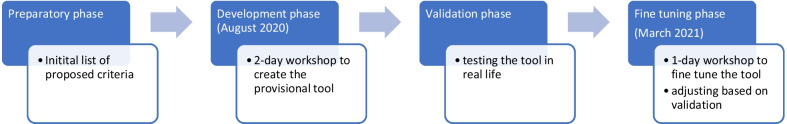
- MCDA tool development process

The MCDA tool was developed in a stepwise approach in four phases (see Fig. 1) according to the guidance by Inotai et al.,^xx^ including (1) a preparatory phase to explore different criteria from previous MCDA tools; (2) a development phase to choose the relevant criteria, determine the scoring functions and weights for each criterion; (3) a validation phase to test the tool and assess its validity and reliability in real life; (4) and a final phase to fine-tune the tool prior to its formal use in tendering process according to the validation findings.

### Preparatory phase

A literature review was conducted to identify the criteria used for purchasing off-patent pharmaceuticals in previous tools. We searched scientific publications as well as grey literature, including local reports, previous evaluation frameworks and MCDA tools that were used in Egypt for similar purposes. For scientific publications, Medline was searched through PubMed, and for grey literature, ISPOR presentations database was searched. Additionally, Google search engine was used to find local reports or unpublished MCDA tools. The search was conducted in June 2019 and the timeframe was not limited.

Studies which included MCDA tools or value frameworks with specific decision criteria mentioned were considered relevant. Snowballing technique was also utilized to locate relevant MCDA tools or value frameworks from the included studies. The search term used in all searches was ((“multi- criteria decision analysis” OR MCDA OR “value framework” OR “multi criteria decision analysis”) AND (criteria OR criterion OR attribute*)).

Relevant criteria for purchasing off-patent pharmaceuticals were identified mainly from three different studies [20], in addition to scoring tools previously used in tenders conducted by several Egyptian universities and institutions (Cairo University, Ain Shams University, Zagazig University, Ministry of Health tender, Shefaa El Orman Oncology Hospital & National Cancer Institute). Criteria identified from the literature were consolidated and de-duplicated to develop a primary list of criteria. For each criterion, a scoring function on a scale from 0 to 100% was proposed based on the literature findings.

During the preparatory phase, unstructured interviews were conducted with public procurement experts in Egypt to confirm the validity of the proposed criteria and identify specific indicators that can objectively measure performance in each criterion (scoring functions).

Due to the policy and internal regulations of the tender process in Egypt, acquisition cost could not be directly included in the MCDA criteria, so only technical components were included in the tool. The technical committee should provide a score on a scale from 0 to 100 points for each medicine using the MCDA tool, then the results for all off-patent alternatives are sent to the financial committee with a suggestion to choose the option which provides the lowest price per point.

*E.g., If a medicine scores 80 points in MCDA and has a price of 160 EGP, and another medicine with the same active product ingredient scores 70 points for 150 EGP, then the first medicine costs 2 EGP per point and the second medicine costs 2.14 EGP per point, so the better option is the first medicine.*

### Development phase

A 2-day workshop was conducted in August 2020 in Cairo aiming to create a provisional MCDA tool. Multiple stakeholders representing organizations for whom the UPA conducts central tendering were invited to participate in developing the MCDA tool.

#### Workshop introduction

During the workshop, participants were introduced to MCDA concept and methodology in addition to the primary proposed list of criteria. Importance of different criteria and their validity in the current local settings were discussed. Participants were asked to vote for potential modifications to the criteria, inclusion of additional criteria, criteria ranking, scoring functions, and relative weight of each criterion. Voting was conducted anonymously by using the Mentimeter® software. To calculate average weights and scores, median values of the votes were used rather than mean values to minimize the effect of outlier votes.

#### Criteria

Starting from the primary list of 13 criteria, participants were asked about the inclusion of additional criteria, potential modifications, or exclusion of any of the criteria. If any modification was requested, voting was done, and if more than half the participants agreed, the modification was implemented. Abiding by the good practice recommendations,xx the complexity of the tool was reduced by choosing less than 10 criteria in the final tool, which enables the significance of each criterion on the final decision.

#### Ranking the criteria

Participants were asked to rank the chosen criteria according to their relevant importance through the voting system. For the final ranking, the average of all votes was calculated.

#### Scoring functions

The performance of an assessed medicine is evaluated in each criterion, and it receives a score from 0 to 100% based on possible outcomes achieved. For each criterion, there was a proposed scoring function based on the literature search, however, participants had the right to change the proposed scoring functions. Additionally, they were asked to provide a score for each possible outcome for the criteria.

*E.g., Participants were asked to provide a score for a medicine about the macroeconomic benefit it provides. The options were having no manufacturing activity in Egypt, having only local packaging in Egypt or having full manufacturing in Egypt. Each participant voted for a score for each of the 3 options on a scale from 0 to 100%, where 0% has the least macroeconomic benefit and 100% has the largest macroeconomic benefit.*

#### Weighting

To assign a relative weight for each criterion, the SMART (simple multi-attribute rating technique) method combined with swing-weighting technique was used to determine the importance of each criterion compared to the next [21]. In this method, participants vote for how much more important one criterion was compared to another. For example, how much more important is the 6th ranked criterion compared to the 7th? followed by how much more important the 5th criterion is compared to the 6th? and so on, until reaching the 1st criterion. Weights were then aggregated and normalized to a total weight of 100, so each criterion would have a specific weight in the final tool summing to 100.

#### Minimum threshold

To ensure the quality of the products that qualify for the financial assessment phase, a minimum score (threshold) was voted upon during the workshop. Achieving a score below the threshold indicates unacceptable quality according to participants’ consensus and will lead to exclusion from the tender.

#### Final score and provisional MCDA tool

To calculate the final score for a medicine, for each criterion, the score achieved is multiplied by the corresponding weight of the criterion to calculate the final weighted score for each criterion. Weighted scores are then summed to reach the final score of the medicine. Based on the voting results, a provisional version of the MCDA tool was developed to be tested and validated.

### Validation phase

Following the workshop, members of the UPA and clinical experts tested the provisional tool version on three real cases of off-patent oncology medicines. Accordingly, they created a list of comments and proposed amendments to be discussed in the final workshop in the presence of all the stakeholders.

### Final (fine-tuning) phase

A one-day workshop was held in March 2021 to review the tool after being tested and to develop a final version for the formal use by UPA. During the workshop, results of real cases were presented and issues with the tool were highlighted. Results of the test cases were discussed, then participants voted for the required amendments. The tool was then fine-tuned according to the voting results.

## Results

### Literature review and preparatory phase

The literature review identified a broad list of 54 criteria with significant overlaps. After a consolidation and deduplication process, the primary list was reduced to 13 criteria. Table 1 provides a summary on the primary criteria list with a brief description on their rationale.

### Development phase

The developed MCDA tool with the final adjustments, ranking, weights and criteria are presented in this section.

#### Workshops participants

Thirty-five experts specialized in tenders and purchasing pharmaceuticals from several governmental entities attended the workshops and participated in voting. The participants were representatives of UPA, Egyptian Drug Authority (EDA), Ministry of Health and Population (MoHP), Egyptian university hospitals, Curative Care Organization (CCO), Health Insurance Organization (HIO), Specialized Medical Centers (SMC), Teaching Hospitals and Institutes Organization (THIO) and other governmental decision-makers from healthcare facilities.

#### Criteria

During the first workshop, the participants modified the initial list of criteria for different reasons. The “refund or replacement of expired products” criterion was excluded, since providing this service is mandatory for tender requirements. The “active pharmaceutical ingredient manufacturing quality” and “finished product manufacturing quality” criteria were merged into a single criterion named “manufacturing quality” to avoid redundancy. The “expiry date”, “ease of use” and “storage conditions” criteria were excluded because pharmaceuticals usually have similar scores for these criteria and such redundancy would not provide true differentiation. As for the “production capacity and financial ability of the company” criterion, it was excluded as participants agreed that it is already reflected in the “supply reliability” criterion and would be an overlap. Subsequently, the initial list of 13 criteria was reduced to seven criteria.

#### Ranking

The selected criteria (seven criteria) were ordered and ranked according to their importance based on voting as follows: (1) use in reference countries; (2) equivalence with the reference product (or reference product); (3) manufacturing quality; (4) pharmacovigilance services; (5) supply reliability; (6) previous use in local settings; (7) macroeconomic benefit.

#### Scoring functions

The scoring functions for each criterion are presented in Table 2, with each criterion having several options for fulfillment of the criterion. Consensus was reached to consider certain scores as exclusion reasons, like medicines not marketed in the country of origin, medicines lacking a GMP certificate or medicines with supply reliability less than 25% (i.e., 75% or more of its orders are late or incomplete). Products with any of the above exclusion reasons, will be excluded from the tender process irrespective of fulfilling any of the other criteria. Table 2 shows the final list of criteria and their scoring functions based on the voting results.

The scoring functions were applied to all criteria except for one, the “pharmacovigilance services” criterion. Instead of receiving a score based on the fulfillment of possible scoring elements, an agreement was reached to use the score provided by the pharmacovigilance administration in the Egyptian Drug Authority. The administration will provide UPA with a report concerning the quality of the pharmacovigilance services provided for the requested medicines. The report would include a final score presented as a percentage fulfillment of pharmacovigilance services required.

#### Criteria weights

Relative weight for each criterion was assigned based on the participants’ votes using the SMART swing-weighting technique. Table 3 shows the final weights for all criteria. The first ranked criterion “use in reference country” had the highest weight of 23.49%, while the last criterion had a weight of “8.67%”.

#### Threshold

A minimum score of 65 points was agreed on to pass the MCDA tool and become eligible for the financial phase of the tender.

### Validation phase

A “60 point” threshold was voted for in the first workshop, however, after testing the tool on real cases, the threshold was revisited and during the second workshop, the majority (9 of 16 participants), voted for a “65 point” threshold. Participants have also voted for exclusion of products which have a supply reliability of less than 25% from the tender instead of giving it a low score for that. The criteria weights were also fine-tuned by the participants. Before validation, the first criterion weighed 35.5% and the last weighed 2%. This provided a huge effect for the first criterion and a negligible effect for the last one. Participants amended this, and the final tool had closer values that ranged from 8.67% to 23.49%.

### Final tool

After the final amendments were implemented, the final version of the tool was shared with UPA and other governmental stakeholders to be used in the tendering process. The final MCDA tool is a Microsoft Excel form that has a dropdown list for different scoring elements of medicines, it automatically calculates the score for each medicine based on the inputs provided and compares performance using values and figures.

## Discussion

The aim of this research was to create an MCDA tool for national tenders of off-patent oncology medicines in Egypt. We created an MCDA tool based on the votes of different governmental bodies’ representatives. The created tool assesses the technical aspects of the available off-patent medicines and provides a final score for each medicine. Each medicine accumulates the score based on 7 predefined criteria in the tool. Medicines which aggregate a score of 65 or more are eligible for the financial phase of the tender to choose the product/s which provide the lowest price per point.

With more than 120 local pharmaceutical companies on top of multinational companies [22–24], several alternatives exist for most of the commonly used active pharmaceutical ingredients in Egypt. Availability of alternatives for the same molecules challenges decision-makers to favor between different competitors. This problem is more obvious when dealing with expensive products as oncology medicines. Currently, the decision is based mainly on price and subjective expert opinion, which was proven by practice not to be the preferred method. Using a transparent MCDA tool allows decision-makers to take unbiased decisions based on evidence related to multiple policy-relevant attributes. The MCDA tool allows for an objective and reproducible comparison between multisource oncology medicines for tendering purposes. Using MCDA will not only support objective decision-making in tendering, but will also act as an incentive scheme for manufacturers to improve the aspects that matter the most for the payer. When the tool is implemented, a manufacturer might be incentivized to seek international GMP certification, maintain a clean supply penalty record, invest in local manufacturing capacity, or improve the provided pharmacovigilance services to achieve a higher score in the MCDA tool.

Another advantage of implementing MCDA tools and value frameworks in decision-making is that since it is conducted based on the collective opinion of multiple stakeholders with potentially different perspectives, it aggregates the various perspectives into one single tool and can usually fit in all settings.

Each MCDA tool is developed based on local settings and needs, so probably no two MCDA tools are identical. However, some similarities can be observed between the newly developed and previously published tools. Our tool shares three similar criteria with the version developed in Indonesia for purchasing off-patent pharmaceuticals: “the equivalence to the reference product”, “pharmacovigilance services”, and “supply reliability”.4 The Indonesian tool is also aligned with the newly developed Egyptian tool in the “quality assurance” criterion, which corresponds to the “manufacturing quality” criterion in our tool. In the same context, an MCDA tool used in Thailand13 encompasses three similar criteria with our tool, namely “macroeconomic benefit”, “supply reliability”, and “manufacturing quality”. However, weights differ between the tools, as each tool is designed for a different decision problem.

Our study had certain limitations. Concerning the criteria, not all MCDA tools or value frameworks used globally were published, so we had to search for grey literature and unpublished reports. This may have led to missing some criteria that were used in unpublished MCDA tools. However, the expert interviews conducted, continuous discussions, and brainstorming sessions have probably covered the majority of policy-relevant aspects of choosing between off-patent medicines.

Due to the two-phase tendering process with the technical and financial committees working independently, it was a challenge to implement MCDA in Egypt. The use of the price per point concept to overcome that issue preserves the MCDA concept; however, it does not allow for the direct control of the price weight.

Although the developed tool went through a pilot testing for validation and was revised accordingly, it should not be considered unchangeable. Instead, the proposed tool should be considered dynamic, with regular workshops held at reasonable intervals (e.g., every 1–2 years) to revisit and fine-tune the tool based on decision-makers’ experiences as well as market dynamics.

## Conclusion

A user-friendly MCDA tool that provides an immediate overall score for each off-patent oncology medicine assessed was developed. The transparent scoring functions and relative weights of each of its seven criteria allow for transparent and reliable scoring of medicines, whereas the price per point concept adapts with the UPA tender policy and can be easily used to identify the appropriate medicines to purchase.

In summary, the proposed MCDA tool is capable of objectively comparing similar off-patent oncology medicines. It reduces subjectivity and provides evidence-based, transparent, and reproducible purchasing decisions. It may also incentivize suppliers and manufacturers to improve their products and services.

## Appendix

See Fig. 1 and Tables 1, 2, 3.

**Table 1:** Primary list of criteria

Criterion	Rationale of the criterion
Use in reference countries	To assure approval from other trusted marketing authorization bodies
Equivalence with the reference product	To capture evidence on equivalence with originator product (bioequivalence, efficacy, safety)
Active pharmaceutical ingredient (API) manufacturing quality	To capture evidence on API manufacturer quality and standardization
Finished product manufacturing quality	To capture evidence on pharmaceutical product manufacturer quality and standardization
Pharmacovigilance services	To capture evidence on provision of proper pharmacovigilance services
Supply reliability	To capture reliability and stability of drug supply (to avoid shortages)
Previous use in local settings	To consider previous local insights and experience with the product
Macro-economic benefit	To capture wider economic benefits of selecting the medicine (e.g., tax, investment, employment, etc.)
Ease of use	To assure ease of use and convenience from the healthcare facility perspective
Expiry date	To incentivize products with high stability and further expiry ranges
Storage conditions	To incentivize products that do not require special storage conditions
Refund or replacement of expired products	To incentivize manufacturers who provide refunds or replacements of expired products
Production capacity and financial ability of the company	To ensure the manufacturer’s ability to supply the required quantities without shortages and on time

**Table 2:** Final list of criteria with scoring functions

Criterion name	Possible scoring elements	Score (%)
Use in reference countries	Pharmaceutical has an FDA or EMA certificate	100%
	Pharmaceutical is used in any of the reference countries	70%
	Pharmaceutical is not used in any reference country and doesn't have FDA or EMA certificate	20%
	Pharmaceutical is not marketed in its country of origin	EXCLUSION
Equivalence with the reference product	Bioequivalence is proven through clinical trials or real-world evidence OR reference product	100%
	Intravenous dosage form OR bioequivalence proven through FDA or EMA	80%
	Local bioequivalence certificate (Only for non-intravenous dosage forms)	50%
	Pharmaceutical equivalence certificate only	20%
Manufacturing quality	WHO or PIC/s GMP certificate for both API and finished product	100%
	WHO or PIC/s GMP certificate for API or finished product and the other certificate from another source	70%
	GMP certificate for both API and final product from another source than WHO or PIC/s	40%
	No GMP certificates	EXCLUSION
Pharmacovigilance	A percent of pharmacovigilance measures fulfillment to be provided for each product based on the pharmacovigilance unit report	(0%–100%)
Supply reliability	No supply reliability issues during the previous 2 years	100%
	Supplier has 75% or more reliable supplies during the previous 2 years	80%
	Supplier has 50%—< 75% reliable supplies during the previous 2 years	60%
	Supplier has 25%—< 50% reliable supplies during the previous 2 years	30%
	No previous supply	20%
	Less than 25% supply reliability during the previous 2 years	EXCLUSION
Previous use in local settings	Pharmaceutical is in the local market for 5 or more years, and has won 4 or more governmental tenders during the last 3 years	100%
	Pharmaceutical is in the local market for less than 5 years, and has won 4 or more governmental tenders during the last 3 years	80%
	Pharmaceutical is in the local market for 5 or more years, and has won less than 4 governmental tenders during the last 3 years	60%
	Pharmaceutical is in the local market for less than 5 years, and has won less than 4 governmental tenders during the last 3 years	20%
	Pharmaceutical is in the local market for less than 3 years, and has not won any governmental tender during the last 3 years	0%
Macroeconomic benefit	The company has full manufacturing in Egypt	100%
	The company has only a packaging factory in Egypt	70%
	The company has no factory in Egypt	30%

*FDA* Food and Drug Administration, *EMA* European Medicines Agency, *WHO* World Health Organization, *GMP* Good Manufacturing Practice, *PIC*/s Pharmaceutical Inspection Co-operation Scheme, *API* active pharmaceutical ingredient

The final list included seven criteria used in the MCDA tool with a scoring function for each criterion. Each product is assessed based on these criteria, and it receives a score for each based on its performance in the criterion

**Table 3:** Criteria weights

#	Criteria	Weight (%)
1	Use in reference countries	23.49
2	Equivalence with the reference product (or reference product)	18.79
3	Manufacturing quality	15.53
4	Pharmacovigilance services	12.94
5	Supply reliability	10.78
6	Previous use in local settings	9.80
7	Macro-economic benefit	8.67

Based on SMART swing weighting, each criterion had a relative weight to participate in the final aggregated score. The weights sum up to 100%. If a product had the best performance for a criterion, it would receive the criterion’s total weight

## Abbreviations

API: Active pharmaceutical ingredient
CCO: Curative Care Organization
EDA: Egyptian Drug Authority,
EGP: Egyptian pounds
EMA: European Medicines Agency
FDA: Food and Drug Administration
GMP: Good Manufacturing Practice
HIO: Health Insurance Organization
MCDA: Multi-criteria decision analysis
MoHP: Ministry of Health and Population
PIC/s: Pharmaceutical Inspection Co-operation Scheme
SMART: The simple multi-attribute rating technique
SMC: Specialized Medical Centers
THIO: Teaching Hospitals and Institutes Organization
UPA: The Egyptian Authority for Unified Procurement, Medical Supply, and The Management of Medical Technology
WHO: World Health Organization
